# A Comparison of Key Essential Nutrients in Commercial Plant-Based Pet Foods Sold in Canada to American and European Canine and Feline Dietary Recommendations

**DOI:** 10.3390/ani11082348

**Published:** 2021-08-09

**Authors:** Sarah A. S. Dodd, Anna K. Shoveller, Andrea J. Fascetti, Zengshou Z. Yu, David W. L. Ma, Adronie Verbrugghe

**Affiliations:** 1 Department of Clinical Studies, Ontario Veterinary College, University of Guelph, Guelph, ON N1G 2W1, Canada; sdodd@uoguelph.ca; 2 Department of Population Medicine, Ontario Veterinary College, University of Guelph, Guelph, ON N1G 2W1, Canada; 3 Department of Animal Biosciences, University of Guelph, Guelph, ON N1G 2W1, Canada; ashovell@uoguelph.ca; 4 Department of Molecular Biosciences, School of Veterinary Medicine, University of California, Davis, CA 95616, USA; ajfascetti@ucdavis.edu (A.J.F.); zzyu@ucdavis.edu (Z.Z.Y.); 5 Department of Human Health and Nutrition Sciences, University of Guelph, Guelph, ON N1G 2W1, Canada; davidma@uoguelph.ca

**Keywords:** balanced diet, canine nutrition, cat food, dog food, feline nutrition, nutritional adequacy, vegan, vegetarian

## Abstract

**Simple Summary:**

Plant-based pet foods appear to be growing in popularity, but it is unclear how suitable these products are for dogs and cats, considering both species naturally consume diets rich in, or exclusively comprised of, animal tissues. Laboratory analyses of essential nutrients were performed on 26 plant-based diets available in Ontario, Canada in 2018, including 18 canine products (13 products labelled for adult maintenance, four for all life stages, one for puppy growth), 5 feline products (two for adult maintenance and three for all life stages), and 3 products labelled for both dogs and cats (one for adult maintenance and two for all life stages). The nutrient measurements were compared to industry standard nutrient profiles. Four products met the recommendations of the Association of American Feed Control Officials and one product the nutrient recommendations of the European Pet Food Industry Federation for adult dogs. No diets met recommended nutrient content for adult cats or growing puppies or kittens. Nutrients most commonly found to be insufficient were: sulfur amino acids, taurine, arachidonic acid, EPA and DHA, calcium phosphorus and vitamin D. These nutrients are all typically found in animal tissues, though non-animal sources are available, and require careful formulation or supplementation in products made without animal-derived ingredients. Compliance with Canadian labelling regulation and guidelines was poor. These problems do not appear to be exclusive to plant-based foods and have been demonstrated previously in commercially available animal-based products as well. This study demonstrates areas where manufacturers of plant-based pet foods must improve formulation and/or manufacturing practices to produce products appropriate for feeding cats and dogs.

**Abstract:**

Plant-based foods intended for feeding dogs and cats are available in Canada, though few studies have examined the suitability of plant-based foods for dogs and cats. All commercial plant-based extruded and wet pet food products available in Ontario, Canada, in 2018 (n = 26) were acquired and analysed for energy, crude protein, crude fat, crude fibre, ash, amino acids, fatty acids, minerals and vitamins A, B_12_, D_2_ and D_3_. Results were compared with recommendations of the Association of American Feed Control Officials (AAFCO) and the European Pet Food Industry Federation (FEDIAF). Thirteen products were labelled for adult canine maintenance, four for canine all life stages, one for puppy growth, two for adult feline maintenance, three for feline all life stages, one for adult maintenance of dogs and cats and two for all life stages of dogs and cats. Four products met AAFCO and one product met FEDIAF nutrient recommendations for canine maintenance. No diets met AAFCO or FEDIAF recommendations for feline maintenance or growth for either species. Nutrients most commonly found insufficient were: sulfur amino acids, taurine, arachidonic acid, EPA and DHA, calcium phosphorus and vitamin D. There were no nutrients unable to be provided from non-animal sources. Compliance with labelling guidelines was also poor, similar to other findings with commercial animal-based pet products. The results from this study indicate areas where producers of plant-based pet foods must improve to meet the industry recommended nutrient profiles and labelling requirements.

## 1. Introduction

Commercial pet foods are often formulated to meet specific target nutrient profiles and exceed minimum nutrient requirements for the species and life-stage for which the product is intended.

In the USA, recommended nutrient profiles are published by the Association of American Feed Control Officials (AAFCO) [1]. In European countries, the European Pet Food Industry Federation (FEDIAF) is the trade body representing European pet food manufacturers, which collaborates with European Union regulatory authorities to play a similar role as AAFCO within the pet food industry [2]. Canada does not have a regulatory agency overseeing pet food specifically, instead, Canadian manufacturers may voluntarily join the Pet Food Association of Canada and adopt AAFCO recommendations [3].

Plant-based pet foods, fed to around 10% of dogs and 3% of cats [4], may be at particular risk of nutrient insufficiencies. One US study detected amino acids below AAFCO recommendation in two commercially available plant-based feline foods [5]. One of the foods also had multiple mineral and vitamin insufficiencies, the other had one analysed vitamin below recommendation [5]. More recently, another study in the USA found that 75% of 24 examined vegetarian pet foods met all minimum AAFCO amino acid recommendations, but only one third met labelling regulations [6]. Of note was that all canine, but few feline foods, met minimum adult maintenance amino acid recommendations. Four Brazilian products met minimum AAFCO and FEDIAF macronutrient recommendations, though only two met recommendations for all analysed amino acids, fatty acids and minerals [7]. All products tested did not meet the appropriate calcium to phosphorus ratio.

Though vitamin D has not been a focus of previous nutrient analyses studies, provision of this vitamin in particular warrants specific consideration. Vitamin D can be provided in two dietary forms: cholecalciferol (vitamin D_3_), found predominantly in animal tissues, or ergocalciferol (vitamin D_2_), found exclusively from non-animal sources [8]. In cats, it has been demonstrated that dietary vitamin D_2_ does not have the same efficiency as vitamin D_3_ to maintain plasma 25-hydroxyvitamin D [9], the main circulating vitamin D metabolite [10,11,12]. In dogs, there is little comparable research, with some confusion remaining regarding the suitability of ergocalciferol as a source of vitamin D for dogs [12]. Nevertheless, plant-based diets may rely on non-animal derived ergocalciferol. Cholecalciferol has been detected in lichen species, which may serve as a source of dietary vitamin D_3_ in plant-based pet foods [13].

Considering the comparatively high prevalence of veganism reported within Canada [4], feeding plant-based diets may be more common than in other countries. However, the nutritional sufficiency of plant-based foods commercially available in Canada has not been investigated. Considering the nutrients identified to be potentially problematic in previous studies, the objective of this study was to measure essential nutrients of particular concern, including protein, amino acids, fat, fatty acids, minerals and vitamins A, B_12_ and D_2_ or D_3_ in all the plant-based pet foods commercially available in Canada and compare to AAFCO and FEDIAF nutrient recommendations for dogs and cats; as well as to assess compliance with pet food label requirements. It was hypothesized that products would comply with the labelling regulations and that they would meet or exceed all nutrient recommendations of the industry organisations. Furthermore, it was expected that North American products may contain vitamin D_2_, though European products would only contain vitamin D_3_ as that is required to meet FEDIAF recommendations.

## 2. Materials and Methods

All twenty-six plant-based pet foods commercially available in Ontario, Canada in 2018 were included in the study. Manufacturers (V-Planet, Petcurean, Halo, Natural Balance and VGRRR) and a retail distributor (Vecado) were contacted by email with an invitation to participate. The letter included a description of the analyses to be performed and a request for donation of products for analyses. Participating companies received a nutrient analysis report for their own products prior to submission for publication. All products were procured at the same time. Six products were donated, 18 provided at cost and two purchased outright. Dry samples were collected from a single bag, wet samples were collected from a single can, all from the same batch. Bags were opened, samples immediately collected from multiple parts of the bag and combined, then analysed or shipped to analytical laboratories within 24 h. Additional samples were acquired at the time of bag opening, vacuum sealed, and frozen at −20 °C. Cans were transported to the laboratories unopened and opened and sampled immediately prior to analyses. Additional cans were left unopened and stored at room temperature.

### 2.1. Proximate Analyses

Product samples were sent to the Central Testing Laboratory in Winnipeg, Manitoba, Canada. Dry matter, gross energy, crude protein, crude fibre, crude fat and ash were measured, moisture, metabolizable energy, and nitrogen-free extract (an approximation of carbohydrates), were calculated. Protein was determined by the Dumas method [14,15]. Crude fat was determined by ether extract (AOAC 954.02) [16]. Crude fibre, a measure of most insoluble fibres, was determined by acid (sulfuric acid) and alkaline (potassium hydroxide) digestion (AOAC 962.09) [16]. Nitrogen-free extract was calculated by subtraction of crude protein, crude fat, crude fibre and ash from the total dry matter, and expressed as %DM. Gross energy (GE) was calculated by the equation: GE = (5.7 ∗ protein) + (9.4 ∗ fat) + 4.1(NFE + fibre) [8]. Metabolizable energy (ME) for dogs was calculated by the equation: ME = 575 + 0.816 ∗ GE(kcal/kg) + 12.08 ∗ fat − 52.76 ∗ crude fibre − 20.61 ∗ protein − 6.06 ∗ moisture. For cats, ME = (GE ∗ energy digestibility/100) − (0.77 ∗ protein) [8]. According to the laboratory, the analyses had a variance of 0.3% for crude protein and 0.5% for crude fat.

### 2.2. Amino Acid Analyses

Food samples were sent to the Amino Acid Laboratory at the School of Veterinary Medicine of the University of California, Davis, in Davis, CA, USA. Detailed description of methodology can be found for amino acid analysis (other than tryptophan) by AOAC official method 994.12, and for tryptophan by AOAC official method 988.15 [16]. Norleucine was used as an internal standard. With acidic hydrolysis, recovery of cystine was about 50% and results were corrected by multiplication factor. Methionine recovery was between 98–102%. According to the laboratory, the amino acid analyses had a coefficient of variation within 5. All essential amino acids: arginine, histidine, isoleucine, leucine, lysine, methionine + cystine, phenylalanine + tyrosine, taurine (essential within the diet for cats, not dogs), threonine, tryptophan and valine were included in the study.

### 2.3. Fatty Acid Analyses

Food samples were analyzed at the University of Guelph for fatty acids. Samples were prepared as-is, with dry kibbles being processed into a fine powder and wet foods being homogenized prior to analysis. Briefly, lipids were extracted by organic solvent in chloroform and methanol, then fatty acids were saponified and methylated using boron trifluoride Methylated fatty acids were separated and analyzed by gas chromatography [17]. C17:0 was added as an internal standard for quantification. Samples were analysed in duplicate and the mean of the two results reported. Dietary contents of all essential fatty acids: linoleic acid, α-linolenic acid (ALA), and long chain metabolites including arachidonic acid, eicosapentaenoic acid (EPA) and docosahexaenoic acid (DHA) were included in the study. In addition, results for γ-linolenic acid (GLA) are also reported, as it has been demonstrated that dietary GLA is effective at maintaining plasma and red blood cell arachidonic acid in adult cats fed arachidonic acid-deficient diets [18,19].

### 2.4. Mineral Analyses

Food samples were sent to the Central Testing Laboratory in Winnipeg, Manitoba, Canada. Mineral evaluation was performed by multi-element inductively coupled plasma optical emission spectrometry (ICP-OES) aka atomic emission spectroscopy (ICP-AES) dry ash method (AOAC 985.01) [16].

### 2.5. Vitamin Analyses

Food samples were sent to Bureau Veritas in Mississauga, Ontario, Canada. Detailed methodology for Vitamin A, retinol and beta-carotene analysis can be found in AOAC official method 992.04 and 992.06, for vitamin B12 in AOAC official method 986.23, and vitamin D3 in AOAC official method 982.29 [16]. For canine products, vitamin A content was determined from vitamin A measured in the product as retinol as well as beta-carotene, as dogs are able to cleave carotenoid precursors into active vitamin A. Thus, both retinol and beta-carotene contribute to total vitamin A intake [8]. For feline products, vitamin A content was determined from vitamin A measured in the product as retinol only, as cats are unable to cleave carotenoid precursors into active vitamin A [8]. Similar to vitamin A, vitamin D2/3 was also measured by HPLC-UV. Mean variance was 12% of the reported values. In addition to the total dietary vitamin D (the sum of D2 + D3), the values of ergocalciferol and cholecalciferol are also presented individually, as it is not known if ergocalciferol is utilized with the same efficacy as cholecalciferol to maintain vitamin D status [9].

### 2.6. Label Evaluation

Each product was visually examined and photographed (SD), for all elements legally required by the Canadian Consumer Packaging and Labelling Act, as well as the AAFCO Labelling Guidelines [1,20]. Consumer Packaging and Labelling Act requirements include: common or generic name, net weight, manufacturer’s or importer’s contact information. AAFCO Labelling Guidelines include: manufacturer contact information, product name, intended species, list of ingredients, feeding instructions, calorie content, guaranteed analysis, nutritional adequacy statement and intended life stage for which the product is suitable. The guaranteed analysis printed on the label was compared to the ananlyzed values. The list of ingredients was used to determine the source of vitamin D.

### 2.7. Categorization of Plant-Based Pet Foods

The companies were categorized by region of origin, either North America or Europe. For Canadian companies, membership with PFAC was determined by searching the list of PFAC members published on their website [21]. Products were categorized as canine or feline foods, according to the intended species mentioned on the pet food label; products labelled as intended for both species are included in results for both species. Products were also categorized for adult maintenance if labelled for adult maintenance or all life stages; or categorized for growth if labelled for puppy or kitten growth or all life stages. Thus, products labelled for all life stages were represented in both maintenance and growth groups.

### 2.8. Statistical Analyses

Descriptive statistics were reported as median and range of each dietary nutrient. All foods were compared to both AAFCO and FEDIAF nutrient profiles, reported on a unit per 100 g dry matter [1,2]. Products labelled for adult maintenance, or all life stages were compared to the adult recommendations, and products labelled for growth, or all life stages were compared to the growth recommendations. Thus, products labelled for all life stages were represented in both maintenance and growth comparisons. The FEDIAF nutrient profiles for adult maintenance vary in accordance with energy intake, with the lower predicted energy intake requiring a higher nutrient density. The FEDIAF profiles for puppy growth differ between early growth, less than 14 weeks of age, and late growth from 14 weeks of age and older—both were included in the analyses performed. Calcium recommendations vary also based on predicted adult weight of 15 kg or less, and greater than 15 kg. The AAFCO nutrient profile for puppy growth differs in calcium recommendation for growth of puppies with a predicted adult weight of 31.75 kg or less, and greater than 31.75 kg. Comparisons to AAFCO nor FEDIAF could not be performed for: ergocalciferol, cholecalciferol and GLA, regardless of species or lifestage; taurine for dogs; and arachidonic acid and EPA + DHA for adult dogs; as these nutrients are not considered essential for the species and/or life stage.

For comparison to the labelled guaranteed analysis, the analyzed values for each product were compared to the guaranteed value on the product label and reported as percentage of guarantee fulfilled, with allowance for the analytical variations published by AAFCO [1]. For nutrients with a guaranteed minimum content, percentages of label fulfilment should be equal to or greater than 100% to meet or exceed the guaranteed minimum. For nutrients with a guaranteed maximum content, percentages of label fulfilment should be equal to or below 100% to meet or stay below the guaranteed maximum.

The nutrient content of pet food products is not regulated within Canada, thus comparison and discussion of nutrients is with respect to the industry (AAFCO or FEDIAF) recommendations. Government regulation strictly pertains to food safety and labelling. Discussion of labelling compliance is compared to the Canadian Consumer Packaging and Labelling Act.

## 3. Results

### 3.1. Description of Analysed Plant-Based Pet Foods

A summary of the plant-based pet food products is shown in Table S1. In total, 26 plant-based pet food products from eight different companies were included. Of the six North American companies, three were Canadian while three were American. One of the Canadian companies was a member of PFAC while the other two were not. Two companies were European. Eighteen products were labelled specifically for dogs (13 extruded, 5 wet), five specifically for cats (5 extruded), and three for both dogs and cats (3 wet). Of the canine foods, thirteen were labelled for adult maintenance (8 dry, 5 wet), four were labelled for all life stages (4 extruded) and one for puppy growth (1 extruded). Of the feline foods, two were labelled for adult maintenance (2 dry), three were labelled for all life stages (3 extruded) and none for kitten growth. Of the three combination canine/feline foods, all were wet products; one was labelled for adult maintenance and two were labelled for all life stages.

### 3.2. Nutrients in Canine Maintenance Products

Of the 20 products labelled for canine adults or all life stages, 12 were produced in North America and eight in Europe. Four products met all AAFCO recommendations for adult maintenance, one met all FEDIAF recommendations for adult maintenance at a lower energy intake (95 kcal/kg^0.75^) and two met all FEDIAF recommendations for adult maintenance at a higher energy intake (110 kcal/kg^0.75^) (Table 1).

In comparison to AAFCO, almost every product (19/20, 95%) exceeded all crude protein recommendations. One (1/20, 5%) had a crude protein concentration below the industry recommended minimum, but within the analytical variation published by AAFCO [1]. One extruded product (1/12, 8%) had a single amino acid (methionine + cystine) below AAFCO recommendation, while one or more amino acids were low in all eight wet products (8/8, 100%). Of those eight wet products, all (8/8, 100%) were low in methionine + cystine, three (3/8, 25%) in methionine and methionine + cystine, and one (1/8, 13%) in lysine, methionine, methionine + cystine and tryptophan. All products met AAFCO crude fat and essential fatty acid (linoleic acid) recommendations. Fifteen (15/20, 75%) met all mineral requirements; two extruded products (2/12, 25%) contained sodium below AAFCO recommendations, one (1/12, 8%) had sodium, chloride and calcium below AAFCO recommendations and an inverse calcium to phosphorus ratio. One extruded product (1/12, 8%) had a calcium to phosphorus ratio exceeding the recommended maximum (2:1). Two wet products (2/8, 25%) contained insufficient zinc, and one (1/8, 13%) had insufficient calcium and an inverse calcium to phosphorus ratio (0.8:1). Twelve (12/20, 60%) foods were replete in all vitamins measured. Two extruded products (2/12, 17%) had a single vitamin below AAFCO recommendations (A or D) and one (1/12, 13%) contained insufficient vitamins A, B12 and D. Two wet diets (2/8, 25%) were below AAFCO recommendations in one vitamin (B_12_ or D) and three (3/8, 38%) in vitamins B_12_ and D.

In comparison to FEDIAF recommendations for dogs with a lower energy consumption of 95 kcal/kg^0.75^, seventeen (17/20, 85%) products met the total protein recommendation, and seven (7/20, 35%) met all amino acid recommendations. Two extruded products (2/12, 17%) contained insufficient methionine + cystine, while two (2/12, 17%) contained insufficient methionine and methionine + cystine and one (1/12, 8%) contained insufficient methionine + cystine and tryptophan. All wet products (8/8) contained insufficient methionine + cystine, five (5/8, 63%) contained insufficient methionine and methionine + cystine, and one (1/8, 13%) contained insufficient methionine, methionine + cystine, and tryptophan. All products met FEDIAF crude fat and fatty acid recommendations. Fourteen foods (14/20, 70%) met all FEDIAF mineral minimum recommendations. Four extruded products (4/12, 33%) contained insufficient sodium and one (1/12, 8%) insufficient sodium, chloride, calcium, and an inverse calcium to phosphorus ratio. One extruded product had a calcium to phosphorus ratio greater than the FEDIAF recommendation. Two wet products (2/8, 25%) contained insufficient zinc and one (1/8, 8%) insufficient calcium and an inverse calcium to phosphorus ratio. Of the European manufactured diets, three (3/8, 38%) contained minerals in excess of the EU legal limit. Six (6/20, 30%) products contained all vitamins measured above the FEDIAF minimum recommended content. Six extruded products (6/12, 50%) had a single vitamin below FEDIAF recommendations (A, B_12_ or D), one (1/12, 8%) contained insufficient vitamin A and D, and one (1/12, 8%) contained insufficient vitamin A, B_12_ and D. Two wet diets (2/8, 25%) contained a single vitamin (B_12_ or D) below FEDIAF recommendations and three (3/8, 38%) contained both vitamins B_12_ and D below recommendations.

In comparison to FEDIAF recommendations for dogs with a higher energy consumption of 110 kcal/kg^0.75^, nineteen (19/20, 95%) products met the total protein recommendation, and ten (10/20, 50%) met all amino acid recommendations. One extruded (1/12, 8%) and all wet (8/8, 100%) products contained insufficient methionine + cystine. Four (4/8, 50%) wet products also contained insufficient methionine. All products met FEDIAF crude fat and fatty acid recommendations. Fourteen foods (14/20, 70%) met all FEDIAF mineral minimum recommendations. One extruded product (1/12, 8%) contained insufficient sodium, one (1/12, 8%) contained insufficient sodium and an excess calcium to phosphorous ratio, and one (1/12, 8%) insufficient sodium, chloride, calcium, and an inverse calcium to phosphorus ratio. Two wet products (2/8, 25%) contained insufficient zinc and one (1/8, 8%) insufficient calcium and an inverse calcium to phosphorus ratio. Of the European manufactured diets, three (3/8, 38%) contained minerals in excess of the EU legal limit. Nine (9/20, 45%) products contained all vitamins measured above the FEDIAF minimum recommended content. Five extruded products (5/12, 42%) had a single vitamin below FEDIAF recommendations (A, B12 or D) and one (1/12, 13%) contained insufficient vitamins A, B_12_ and D. Two wet diets (2/8, 25%) were below FEDIAF recommendations in one vitamin (B12 or D) and three (3/8, 38%) in vitamins B_12_ and D.

### 3.3. Nutrients in Canine Growth Products

Of the seven products labelled for canine growth or all life stages, six were made in North America, one in Europe. No product met all AAFCO or FEDIAF recommendations for growing dogs (Table 2).

In comparison to AAFCO recommendations, all products (7/7, 100%) met total protein recommendations, though only four (4/7, 57%) products met all amino acid recommendations. One extruded product (1/5, 20%) contained insufficient methionine + cystine, one wet product (1/2, 50%) contained insufficient methionine + cystine while the other (1/2, 50%) contained insufficient methionine + cystine and lysine. All products contained sufficient total fat, but no product met AAFCO EPA + DHA recommendations. Two products (2/7, 29%) met all AAFCO mineral recommendations. One extruded product (1/5, 20%) had only chloride below AAFCO recommendations, one (1/5, 20%) contained insufficient calcium and phosphorus, while one (1/5, 20%) had calcium, phosphorus and zinc below AAFCO recommendations. In both wet products (2/2, 100%) calcium and phosphorus were below AAFCO recommendations, while one wet product (1/2, 50%) also had insufficient sodium and an inverse calcium to phosphorus ratio. No products exceeded the AAFCO maximum recommended concentration of calcium for large or non-large breed dogs. Four (4/7, 57%) products met all AAFCO vitamin recommendations. One extruded product (1/5, 20%) contained insufficient vitamin A, another (1/5, 20%) insufficient vitamin D. One wet product (1/2, 50%) contained insufficient vitamins B_12_ and D.

In comparison to FEDIAF recommendations for growth of puppies less than 14 weeks of age, all products met total protein recommendations, though only four (4/7, 57%) met all amino acid recommendations. One extruded product (1/5, 20%) and both wet products (2/2, 100%) contained insufficient methionine + cystine (2/5, 40%). All products contained sufficient total fat, but no product (0/7, 0%) met FEDIAF EPA + DHA or arachidonic acid recommendations. Two products (2/7, 29%) met all FEDIAF minimum mineral recommendations. Two extruded products (2/5, 40%) contained a single mineral insufficiency (chloride or phosphorus), while one (1/5, 20%) contained both insufficient phosphorus and zinc. Both wet products (2/2, 100%) contained insufficient calcium and phosphorus, with one (1/2, 50%) having an inverse calcium to phosphorus ratio. The single European diet contained copper in excess of the EU legal limit. Four products (4/7, 57%) met all FEDIAF vitamin recommendations. Two extruded products (2/5, 40%) had a single vitamin below FEDIAF recommendations (A or D) while one wet product (1/2, 50%) contained insufficient vitamins B_12_ and D.

In comparison to FEDIAF recommendations for growth of puppies over 14 weeks of age, all products met total protein and all amino acid requirements. All products met FEDIAF total fat recommendations, but no product (0/7, 0%) contained sufficient EPA + DHA or arachidonic acid. Three products met all FEDIAF mineral recommendations; one extruded product (1/5, 20%) contained insufficient chloride, while one (1/5, 20%) contained insufficient phosphorus and zinc. Both wet foods (2/2, 100%) contained insufficient calcium and phosphorus, with one (1/2, 50%) having an inverse calcium to phosphorus ratio. One extruded product (1/5, 20%) and both wet foods (2/2, 100%) contained insufficient calcium in comparison to FEDIAF recommendations for large-breed puppies, though none of the products were specifically labelled for large-breed puppy growth. Four products met all FEIDAF vitamin recommendations. Two extruded products (2/5, 40%) had a single vitamin below FEDIAF recommendations (A or D) while one wet product (1/2, 50%) contained insufficient vitamins B_12_ and D.

### 3.4. Selected Essential Nutrients in Feline Maintenance Products

Of the eight products labelled for feline adult or all life stages, five were manufactured in North America and three in Europe. No product met all AAFCO or FEDIAF recommendations for adult maintenance (Table 3).

In comparison to AAFCO recommendations, all (8/8, 100%) feline products labelled for maintenance or all life stages exceeded crude protein recommendations. Few products (2/8, 100%) met all amino acid recommendations. Two extruded (2/5, 40%) and no wet (0/3, 0%) products met the AAFCO recommended taurine concentrations. All other amino acid recommendations were met by all (8/8, 100%) products. Six (6/8, 75%) products met AAFCO crude fat recommendations, two wet products (2/3, 67%) did not do so. AAFCO Linoleic acid recommendations were surpassed by all products, though few (2/8, 25%) met arachidonic acid recommendations—both were wet products. Six (6/8, 75%) products met all AAFCO mineral recommendations, with one extruded product (1/5, 20%) containing insufficient sodium and one wet product (1/3, 33%) insufficient calcium. Five products (5/8, 63%) met all AAFCO vitamin recommendations. Two extruded products (2/5, 40%) had a single vitamin below AAFCO recommendations (A or D) and one wet product (1/3, 33%) had both vitamins B_12_ and D below recommendations.

In comparison to FEDIAF recommendations for cats fed a lower energy intake of 75 kcal/kg^0.67^, few (2/8, 25%) feline products labelled for maintenance or all life stages exceeded crude protein recommendations. Two extruded products (2/5, 40%) and two wet products (2/3, 67%) contained only taurine below FEDIAF recommendations. One extruded product (1/5, 20%) contained insufficient arginine, phenylalanine + tyrosine, and taurine, and one wet product contained insufficient methionine + cystine and taurine. Six (6/8, 75%) products met FEDIAF crude fat recommendations, two wet products (2/3, 67%) did not do so. Most (6/8, 75%) diets met all FEDIAF fatty acid recommendations, with one extruded (1/5, 20%) and one wet (1/3, 33%) product failing to so. Five (5/8, 63%) products met all FEDIAF minimum mineral recommendations, with one extruded product (1/5, 20%) having a single mineral insufficiency (potassium), and one (1/5, 20%) having three minerals below recommendation (potassium, iron and zinc). One wet product (1/3, 33%) contained insufficient calcium and an inverse calcium to phosphorus ratio. Two European manufactured diets contained minerals in excess of the EU legal limit: one extruded product (1/5, 20%) contained excess copper and one wet product (1/3, 33%) contained excess zinc. Five products (5/8, 63%) met all FEDIAF vitamin recommendations. One extruded product (1/5, 20%) had a single vitamin below FEDIAF recommendations (D) and one (1/5, 20%) had two (vitamins A and B_12_). One wet product (1/3, 33%) had both vitamins B_12_ and D below FEDIAF recommendations.

In comparison to FEDIAF recommendations for cats fed a higher energy intake of 100 kcal/kg^0.67^, all (8/8, 100%) feline products labelled for maintenance or all life stages exceeded crude protein recommendations, and all amino acid requirements, with the exception of taurine. Only two products, both extruded, met taurine requirements. Six (6/8, 75%) products met FEDIAF crude fat recommendations, two wet products (2/3, 67%) did not do so, but all (8/8, 100%) diets met all fatty acid recommendations. Seven (7/8, 88%) products met all FEDIAF minimum mineral recommendations, with one wet product (1/3, 33%) containing an inverse calcium to phosphorus ratio. Sufficiency of vitamin provision was the same as for the recommendations for cats with a lower intake, described in the section above.

### 3.5. Selected Essential Nutrients in Feline Growth Products

Of the eight products labelled for feline growth or all life stages, all produced by two North American companies, none met all AAFCO and FEDIAF recommendations for feline growth (Table 4).

In comparison to AAFCO recommendations, most (4/5, 80%) feline foods labelled for growth or all life stages exceeded crude protein recommendations for kitten growth and development, though only one product (1/6, 17%) met all amino acid recommendations. One extruded product (1/3, 33%) had a single amino acid (methionine + cystine) below AAFCO recommendations and one (1/3, 33%) had four amino acids (methionine, methionine + cystine, phenylalanine + tyrosine, and taurine) below recommendation. Both wet products contained methionine, methionine + cystine and taurine below AAFCO recommended concentrations. Most (4/5, 80%) diets met AAFCO crude fat recommendations, with one wet product (1/2, 50%) failing to do so. No product met all AAFCO fatty acid recommendations, with all three extruded products containing insufficient arachidonic acid, and all five diets containing insufficient EPA + DHA. Two extruded products (2/3, 67%) met all AAFCO mineral recommendations while one extruded product (1/3, 33%) and both wet products (2/2, 100%) contained insufficient calcium and phosphorus. One extruded product also did not meet the AAFCO minimum copper recommendation Two diets (2/5, 40%) met all AAFCO vitamin recommendations. Two extruded products (2/3, 67%) had a single vitamin (vitamin A or D) insufficiency, while one wet product (1/2, 50%) had both vitamins B_12_ and D below AAFCO recommendation.

In comparison to FEDIAF recommendations, all feline foods labelled for growth or all life stages exceeded crude protein recommendations for kitten growth and development. Two products (2/5, 40%) met all FEDIAF amino acid recommendations. One extruded product (1/3, 33%) had four amino acids (methionine, methionine + cystine, phenylalanine + tyrosine, and taurine). Both wet products contained methionine + cystine and taurine below FEDIAF recommended concentrations. One wet product (1/3, 20%) contained insufficient fat. All extruded products contained insufficient arachidonic acid, though both wet products contained sufficient arachidonic acid. No products met FEIDAF EPA + DHA recommendations. Two extruded products (2/3, 67%) met all FEDIAF minimum mineral recommendations while one extruded product (1/3, 33%) and both wet products (2/2, 100%) contained insufficient calcium and phosphorus and one wet product (1/2, 50%) had an inverse calcium to phosphorus ratio. None of the products were made in Europe, so European legal limits for minerals were not considered. One product (1/5, 20%) met all FEDIAF vitamin recommendations. Two extruded products (2/3, 67%) had a single vitamin insufficiency (vitamin A), while one extruded (1/3, 33%) product contained insufficient vitamins A and D. One wet product (1/2, 50%) had vitamins B_12_ and D below FEDIAF recommended concentrations.

### 3.6. Source of Vitamin D

Based on pet food label information, an equal number of products claimed to include vitamin D_2_ as claimed to included vitamin D_3_. Two wet foods (2/8, 25%), produced by the same company, claimed to include a vitamin D supplement, but did not specify if it was vitamin D2 or vitamin D_3_. Despite FEDIAF recommendations being specifically for vitamin D_3_, six (6/11, 55%) European products listed vitamin D_2_ in the ingredient deck, five (5/11, 45%) listed vitamin D_3_. Six (6/15, 40%) North American diets listed vitamin D_2_ in the ingredient deck, seven (7/15, 47%) listed vitamin D_3_, and two (2/15, 13%) listed a non-specific vitamin D supplement.

When analysed, five products (4 extruded, 1 wet) contained vitamin D_2_ while seventeen (14 extruded, 3 canned) contained vitamin D_3_. Six products (2 extruded, 4 wet) contained no detectable vitamin D. Six (6/12, 50%) of the products claiming vitamin D_2_ had no detectable D_2_ but did contain D_3_, while two (2/12, 17%) contained both D_2_ and D_3_. Three (3/12, 25%) contained D_2_ only. One of the products bearing a claim for an unspecified vitamin D supplement contained D_3_, the other contained no detectable vitamin D. Two European diets claiming vitamin D_2_ content (2/6, 33%) contained vitamin D_2_, both also contained vitamin D_3_. Three North American diets claiming vitamin D_2_ (3/6, 50%) contained vitamin D_2_, none contained both vitamin D_2_ and vitamin D_3_.

### 3.7. Labelling

All but two products met all Canadian legal labelling requirements according to the Consumer Packaging and Labelling Act [20]. The two products failing to meet requirements did not include the contact details of the manufacturer. Both products were manufactured by the same company within Canada. Nine products (9/26, 35%) from five companies (5/8, 63%) met AAFCO Labelling Guidelines. Fifteen products (15/26, 58%) from three companies (3/8, 38%) did not meet AAFCO Labelling Guidelines as they did not include a statement of calorie content. Six (6/15, 40%) foods manufactured in the USA bore a statement of caloric density. Only 3 (3/11, 27%) foods manufactured in Europe bore a statement of caloric density. All products produced in North America (15/15, 100%) included a statement of nutritional adequacy for the species and life stage, as substantiated by formulation to meet the targeted nutrient profile. No diet bore a statement of substantiation by feeding trial. The 11 products manufactured in Europe (11/26, 42%) did not have a nutritional adequacy statement as described by AAFCO, but did include a descriptor of the product being “Complete” for the animal species and life stage intended, in compliance with FEDIAF requirements. In comparison to the labelled guaranteed analysis, seven canine products (7/20, 33%) and no feline products (0/7, 0%) met or exceeded all labelled maxima; 16 canine (16/20, 80%) and six feline (6/7, 86%) complied with labelled minima. One canine and one feline product, both manufactured by the same Canadian company, bore no guaranteed analysis on the label. Table 5 shows the percentage of labelled value measured for each nutrient.

## 4. Discussion

While plant-based diets are shown to support health in humans [22,23,24,25], the nutritional requirements of obligate carnivorous cats and facultative carnivorous dogs are quite different. As a result of their evolution consuming most or part of their diet as prey, both cats and dogs have greater dietary requirements for protein and micronutrients typically found in higher concentrations in animal tissues in comparison to humans. Cats have additional nutritional idiosyncrasies common to carnivorous animals [26,27]. Provision of these nutrients in appropriate quantities for dogs and cats without utilising animal-derived ingredients can be a challenge. The formulation and production of commercial extruded and wet plant-based pet foods containing all essential nutrients required by dogs and cats is possible. Of the 26 plant-based foods commercially available in Canada, few (3/26, 12%) were found to meet all nutrient recommendations for the life stage they were intended, when compared to AAFCO or FEDIAF nutrient profiles. Only adult canine maintenance requirements were met, no product met feline or growth recommendations. Each essential nutrient measured could be detected in the plant-based foods, though the products did not all meet the recommended quantities or proportions. Though outside the scope of this study, digestibility and bioavailability of nutrients must also be considered, in addition to provision in appropriate quantities.

In adult canine foods, the most problematic nutrients, those most often failing to meet minimum recommendations, were the sulfur amino acids methionine + cystine and vitamin D. In products labelled for canine growth, calcium, phosphorus and the omega 3 fatty acids EPA + DHA were most limiting. For the feline adult products, sulfur amino acids were sufficient but taurine was not, particularly in the wet products. Additionally, provision of arachidonic acid was often below recommendations. Feline growth products proved the most problematic, commonly demonstrating insufficiencies in methionine, methionine + cystine, taurine, arachidonic acid, EPA + DHA, calcium and phosphorus. Dietary deficiency of most amino acids may be subclinical and go undetected for long periods of time. Though methionine and cysteine play critical roles in protein synthesis and function, as well as methyl donation and antioxidant generation, the signs of deficiency are non-specific and relate to reduction in food intake, weight loss, poor growth and dermatitis [28,29]. Reduced intake and/or bioavailability of methionine and cysteine in dogs may also predispose to insufficient taurine synthesis and result in dilated cardiomyopathy and retinal changes [30]. In cats, taurine deficiency has been clearly associated with dilated cardiomyopathy [31]. Calcium, phosphorus and vitamin D are all involved in skeletal metabolism and thus deficiencies and/or imbalances can predispose to skeletal deformities and pathological fractures, particularly in young, developing animals [32,33,34]. Arachidonic acid is considered an essential fatty acid for adult cats, kittens, and, in Europe, for puppies [1,2]. The essentiality of this omega-6 fatty acid in adult cats, however, has been questioned. With dietary provision of sufficient linoleic acid, with or without GLA, cats may be able to generate sufficient dihomo-GLA to arachidonic acid via delta-5 desaturase [35]. Adult cats fed diets devoid of arachidonic acid but rich in linoleic acid show no signs of arachidonic acid deficiency unless mated [36]. Even during growth, males appear unaffected, but females demonstrate reliance on dietary arachidonic acid to maintain healthy reproductive activity [37]. It has been concluded that arachidonic acid may not be essential for maintenance, growth, or reproduction in male cats, only for reproduction in female cats [35]. Non-reproducing cats fed plant-based diets devoid of arachidonic acid may thus show no overt clinical signs of arachidonic acid deficiency, particularly if the diets are rich in linoleic acid and/or GLA. Nevertheless, without addition of arachidonic acid the products did not meet industry nutrient guidelines.

These findings share some commonalities with other nutrient analyses of plant-based foods performed previously around the world. In Brazil, a limited sample of three plant-based dog foods were noted to have insufficiencies of methionine (1/3, 33%) while the single plant-based cat food evaluated contained no detectable arachidonic acid [7]. In the USA, a focused evaluation of amino acids in 24 plant-based and vegetarian products detected amino acid insufficiencies in six (25%) of the products, with three (3/6, 50%) of those having insufficiencies of methionine, methionine + cystine and/or taurine [6]. Five of the six products were labelled for cats, one was for dogs and cats. Eleven of the products analysed in that study were included in this current study, though direct comparison of results was not possible as means and ranges for all products were presented and individual product results were not published.

An older study in the USA examined amino acids as well as fatty acids and select vitamins and minerals in two feline products [5]. Findings shared with the present study included insufficiencies in methionine (2/2, 100%), methionine + cystine (2/2, 100%), taurine (2/2, 100%), arachidonic acid (2/2, 100%), calcium (1/2, 50%), phosphorus (1/2, 50%), vitamin A (1/2, 50%), and vitamin B12 (1/2, 50%). In that study, the products were named and one of the products was also evaluated in the present study. In comparison to the previous findings, the product currently contained sufficient methionine, methionine + cystine, vitamin A and vitamin B12. However, the product was still below the recommended inclusion concentrations for taurine and arachidonic acid, and a new insufficiency in threonine was detected. Given that over 10 years have passed between studies, changes to the formulation and manufacturing of the product are likely to have occurred. Between the results of previous studies and the findings of the present study, it is clear that although formulation of foods meeting all recommended nutrient targets for both dogs and cats is possible without inclusion of animal products, there is great variability in the nutritional sufficiency of commercially available plant-based pet foods. Failure to meet industry nutrient recommendations has been demonstrated before in Canadian pet foods [38], though not to the same magnitude as demonstrated in this study. Of the commercial foods analyzed in that study, 93% (25/27) met or exceeded AAFCO nutrient recommendations, in comparison to 12% (3/26) in this investigation. Comparison to labelled guaranteed analyses was more consistent, with 33% (9/27) in the previous study meeting all nutrient content claims, compared to 27% (7/26) of the plant-based products. Given that there were no nutrients identified that were unable to be provided from non-animal sources and that there were products that met all nutrient recommendations, it is unclear if the failure to meet industry recommendations is due to the lack of animal ingredients in the products or if this is a reflection of inadequate formulation or manufacturing processes. A range of practice standards for quality assurance and quality control of pet foods exist. A recent study of pet food manufacturers, including 19 meat-based and 10 plant-based pet foods reported that most manufacturers had standards considered acceptable by the authors, with practices for plant-based diets considered slightly superior [39]. The dietary requirements of animals have been established based on essentiality of nutrients, not ingredients, and there are no known nutrients contained exclusively in animal products that cannot be provided from non-animal sources. Trends in the pet food industry tend to be based on ingredients, however, as a result of marketing and trends in human nutrition and philosophies. It is important to keep in mind that the nutritional requirements of dogs and cats differ from those of humans.

Concerningly, there was great discrepancy in this study between the labelled source of vitamin D and the type of vitamin D measured. Though twelve of the diets included a vitamin D_2_ supplement in their ingredient list, only five were found to contain vitamin D_2_. Two also contained vitamin D_3_, which was not listed in the ingredients. In those diets, no vitamin D_3_-rich ingredients (animal or lichen) or vitamin D_3_ supplements were included in the ingredient list. Cross-contamination could be possible if animal-based diets had been manufactured in the same facility, but this would be insufficient to explain the concentrations of vitamin D_3_ in those diets, as they contained the full recommended quantity.

With respect to the most common nutrient insufficiencies detected, those being sulfur amino acids, taurine, arachidonic acid, EPA + DHA, calcium, phosphorus and vitamin D, correction of the insufficiencies would be expected to be relatively simple [40] (Table 6).

Though non-animal sources of nutrients exist, there may be restrictions on the use of these ingredients in pet food products. For example, ergocalciferol is a non-animal derived source of vitamin D in the form of vitamin D_2_. Ergocalciferol is an approved ingredient for use in pet food formulation in North America [1], though in Europe it is not a registered feed additive, necessitating the addition of cholecalciferol, vitamin D_3_ [2]. Similarly, although EPA and DHA from algal sources have been demonstrated as safe and efficacious for pregnancy, lactation and growth for cats and dogs [48,49], despite this, current regulation limits the use of marine microalgae as a source of DHA and other omega-3 fatty acids for canine adult maintenance in North America [1].

A case report was recently published demonstrating adverse health outcome in cats maintained on a plant-based diet. Fantinati and colleagues identified multiple nutrient deficiencies in two cats fed a commercial plant-based product in Toulouse, France [50]. The cats’ clinical signs were attributed mostly to the marked deficiency of folic acid detected on serum analysis, consistent with insufficiency of folate in the diet. Investigations of health status of dogs and cats fed plant-based diets have been unable to detect any clinical abnormalities associated with the unconventional diet [51,52]. With plant-based feeding being a relatively novel trend and the proportion of pets being fed plant-based diets being low [4], it is possible that adverse outcomes have occurred, but simply have not yet been reported.

In most US states, the AAFCO nutritional and labelling model guidelines are adopted by State Feed Control Officials and all pet foods sold within that state must register products with consideration of the recommendations. In Canada, pet food falls under federal regulation, with labelling and advertising regulated by the Consumer Packaging and Labelling Act [20] and the Competition Act [53]. There is no enforcement through product registration, which includes typical analytical results, regarding the nutrient content of pet food products manufactured within the country, with the exception of federal safeguards for specific risk materials outlined in the Safe Food for Canadians Regulations [54]. Nonetheless, the Pet Food Association of Canada (PFAC) [3], a voluntary industry association, requires pet food manufacturer and company members to adopt the AAFCO model bill. Products being imported into Canada must meet the Canadian importation requirements, which primarily focus on the Consumer Acts aforementioned. Pet foods produced and sold exclusively within Canada have been suggested to potentially be at risk of inadequate formulation and misleading labelling [38]. Similar findings have been reported within the USA, with significant differences measured between labelled and analysed nutrient content [55].This is consistent with the findings reported here, with all foods imported into Canada meeting Canadian regulatory requirements, but two products manufactured and sold exclusively within Canada did not do so.

## 5. Conclusions

The findings of the present study suggest that formulation of plant-based foods meeting all nutrient requirements of dogs and cats for all life stages are possible, though current industry practices sometimes fall short of published nutritional recommendations. Concern for the provision of essential nutrients warrants consideration, and testing of products for these common nutrients of concern and/or evaluating their status in pets fed plant-based may be justified. Inclusion of a statement of caloric content is a requirement to meet AAFCO labelling guidelines and would improve compliance of most commercial plant-based pet foods.

## Figures and Tables

**Table 1:** Measured nutrient content in 20 plant-based canine foods commercially available in Canada and labeled for adult or all life stages compared to the AAFCO and FEDIAF recommended essential nutrient concentrations for canine adult maintenance on a dry matter basis (unit per 100 g dry matter).

Nutrient (Unit/100 g DM)	Median	Range	AAFCO *	FEDIAF	
				95 kcal/kg ^0.75^ **	110 kcal/kg ^0.75^ ***
Crude protein (g)	27.6	***17.3***–36.6	18.0	21.00	18.00
Arginine (g)	1.62	1.03–2.57	0.51	0.60	0.52
Histidine (g)	0.60	0.36–0.76	0.19	0.27	0.23
Isoleucine (g)	1.40	0.88–1.65	0.38	0.53	0.46
Leucine (g)	2.41	1.49–5.26	0.68	0.95	0.82
Lysine (g)	1.26	**0.59**–2.09	0.63	0.46	0.42
Methionine (g)	0.52	***0.22***–1.50	0.33	0.46	0.40
Methionine + Cystine (g)	0.83	***0.25***–1.97	0.65	0.88	0.76
Phenylalanine (g)	1.48	1.03–2.24	0.45	0.63	0.54
Phenylalanine + Tyrosine (g)	2.56	1.61–3.89	0.74	1.03	0.89
Taurine (g)	0.11	0.00–0.21	n/a		
Threonine (g)	1.30	0.85–1.56	0.48	0.60	0.52
Tryptophan (g)	0.29	***0.15***–0.38	0.16	0.20	0.17
Valine (g)	1.53	0.96–1.90	0.49	0.68	0.59
Crude fat (g)	13.8	8.5–25.1	5.5		
Linoleic acid (g)	6.41	1.40–17.88	1.1	1.32	1.53
ALA (g)	0.70	0.16–2.13	n/a		
Arachidonic acid (g)	0.01	0.00–0.03	n/a		
EPA + DHA (g)	0.00	0.00–0.05	n/a		
GLA (g)	0.01	0.00–0.07	n/a		
Calcium (g)	1.07	***0.38***–1.90	0.5–2.5	0.58–2.50	0.50–2.50
Phosphorus (g)	0.8	0.5–1.5	0.4–1.6	0.46–1.6	0.4–1.6
Ca:P ratio (g)	1.3	***0.8***–***2.5***	1:1–2:1		
Potassium (g)	1.11	0.73–1.72	0.6	0.58	0.50
Sodium (g)	0.34	***0.02***–1.27	0.08	0.12	0.10
Chloride (g)	0.93	***0.09***–2.47	0.12	0.17	0.15
Magnesium (g)	0.15	0.10–0.23	0.06	0.08	0.07
Iron (mg)	22.76	10.71–*75.04*	4.0	4.17–68.18 (L)	3.60–68.18 (L)
Copper (mg)	2.34	0.89–*5.47*	0.73	0.83–2.80 (L)	0.72–2.80 (L)
Manganese (mg)	4.13	1.39–7.39	0.50	0.67–17.00 (L)	0.58–17.00 (L)
Zinc (mg)	15.52	***4.46***–*35.93*	8.0	8.34–22.70 (L)	7.20–22.70 (L)
Vitamin A (IU)	1001	***274***–3973	500–25,000	702.00–40,000	606.00–40,000
Vitamin D_2_ (IU)	0	0–152	n/a		
Vitamin D_3_ (IU)	73	0–172	n/a		
Total vitamin D (IU)	91	***0***–172	50–300	63.90–227.00 (L)	55.20–227.00 (L)
Vitamin B_12_ (mg)	0.00434	***0.0000***–0.69074	0.0028	0.00387	0.00335

* AAFCO nutrient profile for adult maintenance of dogs (minimum or minimum–maximum) [1]. ** FEDIAF recommendations for adult maintenance of sedentary dogs, for an expected daily energy intake of 95 kcal/kg^0.75^ (minimum or minimum–maximum) [2]. *** FEDIAF recommendations for adult maintenance of active or working dogs, for an expected daily energy intake of 110 kcal/kg^0.75^ (minimum or minimum–maximum) [2]. Bolded values are outside of the AAFCO recommended range. Italicized values are outside of the FEDIAF recommended range for dogs with an energy intake of 95 kcal/kg^0.75^. Underlined values are outside of the FEDIAF recommended range for dogs with an energy intake of 110 kcal/kg^0.75^. AAFCO = Association of American Feed Control Officials, FEDIAF = European Pet Food Industry Federation, NR = No recommendation, (L) = Legal limit.

**Table 2:** Measured nutrient content in seven plant-based canine foods commercially available in Canada and labeled for puppies or all life stage compared to the AAFCO and FEDIAF recommended essential nutrient concentrations for canine growth on dry matter basis (unit per 100 g dry matter).

Nutrient (Unit Per 100 g)	Median	Range	AAFCO *	FEDIAF	
				Early Growth **	Late Growth ***
Crude protein (g)	32.66	28.75–36.64	22.5	25.00	20.00
Arginine (g)	1.69	1.49–1.83	1.0	0.82	0.74
Histidine (g)	0.62	0.57–0.75	0.44	0.39	0.25
Isoleucine (g)	1.41	1.21–1.65	0.71	0.65	0.50
Leucine (g)	2.39	2.05–5.26	1.29		0.80
Lysine (g)	1.30	**0.88**–1.81	0.90	0.88–2.80	0.70–2.80
Methionine (g)	0.56	0.41–1.50	0.35		0.26
Methionine + Cystine (g)	0.86	***0.59***–1.97	0.70		0.53
Phenylalanine (g)	1.51	1.23–2.24	0.83	0.65	0.50
Phenylalanine + Tyrosine (g)	2.50	2.13–3.89	1.30		1.00
Taurine (g)	0.14	0.00–0.20	NR		
Threonine (g)	1.38	1.14–1.53	1.04	0.81	0.64
Tryptophan (g)	0.31	0.26–0.38	0.20	0.23	0.21
Valine (g)	1.54	1.37–1.90	0.68		0.56
Crude fat (g)	13.87	8.54–24.48	8.5		
Linoleic acid (g)	9.04	2.35–*13.89*	1.3	1.30–6.50	1.30
ALA (g)	0.70	0.17–2.13	0.08		
Arachidonic acid (g)	*0.01*	*0.011*–*0.026*	NR	0.030	
EPA + DHA (g)	***0.00***	***0.00***–***0.01***	0.05		
GLA (g)	0.03	0.00–0.05	NR		
Calcium (g)	**1.12**	***0.48***–1.45	1.2–2.5 ^a^	1.00–1.60	0.80 ^a^–1.80
			1.2–1.8 ^b^		1.00 ^b^–1.80
Phosphorus (g)	***0.88***	***0.58***–1.12	1.0–1.6	0.90	0.70
Ca:P ratio	1.3	***0.8***–1.4	1:1–2:1	1:1–1.6:1	1:1–1.8:1 ^a^
					1:1–1.6:1 ^b^
Potassium (g)	1.21	0.73–1.72	0.6	0.44	
Sodium (g)	0.36	**0.29**–0.69	0.3	0.22	
Chloride (g)	1.12	***0.23***–1.41	0.45	0.33	
Magnesium (g)	0.18	0.10–0.67	0.06	0.04	
Iron (mg)	41.53	12.77–61.64	8.8	8.80–68.18 (L)	
Copper (mg)	*3.25*	1.43–*3.49*	1.24	1.10–2.80 (L)	
Manganese (mg)	4.83	1.39–8.26	0.72	0.56–17.00 (L)	
Zinc (mg)	15.61	***9.32***–*35.93*	10.0	10.00–22.70 (L)	
Vitamin A (IU)	965.78	***423.55***–2319.29	500.0–25,000.0	500.00–40,000	
Vitamin D_2_ (IU)	0	0–62	NR		
Vitamin D_3_ (IU)	128	0–165	NR		
Vitamin D (IU)	137.78	***0***–190.04	50.0–300.0	50.00–227.00 (L)	55.20–227.00 (L)
Vitamin B_12_ (mg)	0.00424	***0.00000***–0.69074	0.00280		

* AAFCO nutrient profile for puppy growth (minimum or minimum–maximum) [1]. ** FEDIAF recommendations for growth of puppies up to 14 weeks of age (minimum or minimum–maximum) [2]. *** FEDIAF recommendations for puppies over 14 weeks of age (minimum or minimum–maximum). a. Non-large breed puppies (predicted adult weight up to 31.75 kg [AAFCO] or 15 kg [FEDIAF]). b. Large breed puppies (predicted adult weight over 31.75 kg [AAFCO] or 15 kg [FEDIAF]). Bolded values are outside of the AAFCO recommended range. Italicized values are outside of the FEDIAF recommended range for early growth. Underlined values are outside of the FEDIAF recommended range for late growth. AAFCO = Association of American Feed Control Officials, FEDIAF = European Pet Food Industry Federation, NR = No recommendation.

**Table 3:** Measured nutrient content in eight plant-based feline foods commercially available in Canada and labeled for adult or all life stage compared to the AAFCO and FEDIAF recommended essential nutrient concentrations for feline adult maintenance on a dry matter basis (unit per 100 g dry matter).

Nutrient (Unit Per 100 g)	Median	Range	AAFCO *	FEDIAF	
				75 kcal/kg^0.67^ **	100 kcal/kg^0.67^ ***
Crude protein (g)	*31.61*	*29.16*–35.31	26.0	33.30	25.00
Arginine (g)	1.59	*1.27*–2.45	1.04	1.30	1.00
Histidine (g)	0.63	0.40–0.75	0.31	0.35	0.26
Isoleucine (g)	1.40	0.81–1.61	0.52	0.57	0.43
Leucine (g)	2.39	1.40–4.30	1.24	1.36	1.02
Lysine (g)	1.26	0.88–2.07	0.83	0.45	0.34
Methionine (g)	0.47	0.27–0.85	0.20–1.5	0.23	0.17
Methionine + Cystine (g)	0.68	*0.42*–1.16	0.40	0.45	0.34
Phenylalanine (g)	1.54	0.98–1.87	0.42	0.53	0.40
Phenylalanine + Tyrosine (g)	2.52	*1.55*–3.19	1.53	2.04	1.53
Taurine (g)–extruded	***0.09***	***0.01***–0.21	0.10	0.13	0.10
Taurine (g)–canned	***0.13***	***0.00***–***0.14***	0.20	0.27	0.20
Threonine (g)	1.30	0.73–1.46	0.73	0.69	0.52
Tryptophan (g)	0.33	0.19–0.40	0.16–1.7	0.17	0.13
Valine (g)	1.59	0.94–1.82	0.62	0.68	0.51
Crude fat (g)	10.3	***8.5***–14.6	9.0		
Linoleic acid (g)	5.64	1.40–15.80	0.6	0.67	0.50
ALA (g)	0.68	0.17–1.83	NR		
Arachidonic acid (g)	**0.009**	***0.006***–0.026	0.02	0.008	0.006
EPA + DHA (g)	0.00	0.00–0.01	NR		
GLA (g)	0.01	0.00–0.04	NR		
Calcium (g)	0.84	***0.48***–1.42	0.6	0.53	0.40
Phosphorus (g)	0.79	0.56–1.07	0.5	0.35	0.26
Calcium to phosphorus ratio	1.1	*0.8*–1.4	NR	1:1–2:1	
Potassium (g)	1.06	*0.68*–1.72	0.6	0.80	0.60
Sodium (g)	0.33	**0.17**–0.73	0.2	0.10	0.08
Chloride (g)	0.95	0.38–1.46	0.3	0.15	0.11
Magnesium (g)	0.17	0.09–0.19	0.04	0.05	0.04
Iron (mg)	26.47	*10.49*–42.24	8.0	10.70–68.18 (L)	8.00–68.18 (L)
Copper (mg)	2.49	1.43–*3.44*	0.5	0.67–2.80 (L)	0.50–2.80 (L)
Manganese (mg)	4.11	1.31–6.72	0.76	0.67–17.00 (L)	0.50–17.00 (L)
Zinc (mg)	18.69	*8.90*–*35.93*	7.5	10.00–22.70 (L)	7.50–22.70 (L)
Vitamin A (IU)	907.5	***251.4***–2319.3	333.2–33,330.0	444.00–40,000	333.00–40,000
Vitamin D_2_ (IU)	0	0–26	NR		
Vitamin D_3_ (IU)	103	0–155	NR		
Vitamin D (IU)	111.4	***0***–154.5	28.0–3008.0	33.30–227 (L)	25.00–227 (L)
Vitamin B_12_ (mg)	0.00587	***0.00000***–0.71690	0.0020	0.00235	0.00176

* AAFCO nutrient profile for adult maintenance of cats (minimum or minimum–maximum) [1]. ** FEDIAF recommendations for adult maintenance of indoor and/or neutered cats, for an expected daily energy intake of 75 kcal/kg^0.67^ (minimum or minimum–maximum) [2]. *** FEDIAF recommendations for adult maintenance of active cats, for an expected daily energy intake of 100 kcal/kg^0.67^ (minimum or minimum–maximum) [2]. Bolded values are outside of the AAFCO recommended range. Italicized values are outside of the FEDIAF recommended range for cats eating 75 kcal/kg^0.67^. Underlined values are outside of the FEDIAF recommended range for cats eating 100 kcal/kg^0.67^. AAFCO = Association of American Feed Control Officials, FEDIAF = European Pet Food Industry Federation, NR = No recommendation, (L) = Legal limit.

**Table 4:** Measured nutrient content in five plant-based feline foods commercially available in Canada and labeled for growth or all life stages compared to the AAFCO and FEDIAF recommended essential nutrient concentrations for feline growth on a dry matter basis (unit per 100 g dry matter).

Nutrient (Unit per 100 g)	Median	Range	AAFCO *	FEDIAF **
Crude protein (g)	31.61	**29.16**–35.31	30.0	28.00
Arginine (g)	1.54	1.27–2.45	1.24	1.07–3.50
Histidine (g)	0.61	0.40–0.75	0.33	
Isoleucine (g)	1.39	0.81–1.61	0.56	0.54
Leucine (g)	2.38	1.40–2.77	1.28	
Lysine (g)	1.14	0.88–2.07	1.20	0.85
Methionine (g)	**0.47**	***0.36***–0.85	0.62–1.5	0.44–1.30
Methionine + Cystine (g)	***0.60***	***0.42***–1.16	1.10	0.88
Phenylalanine (g)	1.48	0.98–1.71	0.52	0.50
Phenylalanine + Tyrosine (g)	2.49	***1.55***–2.79	1.92	1.91
Threonine (g)	1.24	0.73–1.40	0.73	0.65
Tryptophan (g)	0.33	0.19–0.38	0.25–1.7	0.16–1.70
Valine (g)	1.54	0.94–1.82	0.64	0.64
Taurine–extruded (g)	0.19	***0.01***–0.21	0.10	
Taurine–canned (g)	***0.13***	***0.00***–***0.14***	0.20	0.25
Crude fat (g)	9.56	***8.50***–14.57	9.00	
Linoleic acid (g)	5.65	1.40–15.80	0.6	0.55
α-linolenic acid (g)	0.68	0.17–1.83	0.02	
Arachidonic acid (g)	***0.01***	***0.01***–0.03	0.02	
EPA + DHA (g)	***0.00***	***0.00***–***0.01***	0.012	0.01
GLA (g)	0.02	0.00–0.04	NR	
Calcium (g)	***0.96***	***0.48***–1.39	1.00	
Phosphorus (g)	***0.76***	***0.56***–1.07	0.8	0.84
Calcium to phosphorus ratio	1.24	*0.83*–1.39	NR	1:1–1.5:1
Potassium (g)	1.44	0.68–1.72	0.60	
Sodium (g)	0.33	0.29–0.73	0.2	0.16
Chloride (g)	1.03	0.79–1.46	0.3	0.24
Magnesium (g)	0.18	0.09–0.19	0.08	0.05
Iron (mg)	24.59	10.49–41.39	8.00	8.00–68.18
Copper–extruded (mg)	*3.14*	**1.46**–*3.44*	1.5	1.00–2.80
Copper–canned (mg)	2.15	1.43–*2.83*	0.84	
Manganese (mg)	4.10	1.31–5.55	0.76	1.00–17.00
Zinc (mg)	21.46	8.90–*35.93*	7.50	7.50–22.70
Vitamin A (IU)	959.5	***251.4***–2319	666.8–33,333	900–33,333
Ergocalciferol (IU)	0.00	0.00–0.00	NR	
Cholecalciferol (IU)	115	0–155	NR	
Vitamin D (IU)	114.7	***0***–154.5	28.0–3008	28.00–227
Vitamin B12 (mg)	0.00464	***0.00000***–0.71690	0.002	0.00180

* AAFCO nutrient profile for kitten growth (minimum or minimum–maximum) [1]. ** FEDIAF recommendations for kitten growth (minimum or minimum–maximum) [2]. Bolded values are outside of the AAFCO recommended range. Italicized values are outside of the FEDIAF recommended range. AAFCO = Association of American Feed Control Officials, FEDIAF = European Pet Food Industry Federation, NR = No recommendation.

**Table 5:** Percentage (%) of label guaranteed fulfilled for each nutrient of the guaranteed analysis in plant-based canine and feline foods commercially available in Canada (n = 26).

	Energy		Crude Protein (min)		Crude Fat (min)		Crude Fibre (max)		Moisture (max)	
	Mean	Range	Mean	Range	Mean	Range	Mean	Range	Mean	Range
Canine extruded	114	102–123	104	82–118	97	66–150	62	22–87	81	56–115
Canine wet	107	102–112	120	92–124	138	21–177	32	21–140	93	90–102
Feline extruded	109	109–109	102	97–119	78	65–86	69	40–102	87	64–120
Feline wet	NR	NR	104	99–106	58	43–82	24	21–27	96	93–98

Min = minimum, Max = maximum, NR = value not reported on product label.

**Table 6:** Provision of key essential nutrients from non-animal sources.

Nutrient	Major Source(s)
Methionine	Synthetic free amino acid [1]
Taurine	Synthetic [1,31]
Arachidonic acid	Algae extract [41,42,43]
EPA	Algae extract [41,42,43]
DHA	Algae extract [41,43]
Calcium	Inorganic minerals [1]
Phosphorus	Inorganic minerals [1] *
Vitamin D	Ergocalciferol [1]; lichen-derived cholecalciferol [44,45]

* Digestibility and bioavailability of inorganic phosphorus should be considered [46,47].

## Data Availability

Data available on reasonable request.

## Supplementary Materials

The following are available online at https://www.mdpi.com/article/10.3390/ani11082348/s1, Table S1: Summary of products.
