# Supplementary material for: A Comparison of Key Essential Nutrients in Commercial Plant-Based Pet Foods Sold in Canada to American and European Canine and Feline Dietary Recommendations
Source: Animals (Basel). 2021 Aug 9;11(8):2348. doi: 10.3390/ani11082348 (PMC8388700; doi:10.3390/ani11082348)
Supplement: Supplementary file 1 [file animals-11-02348-s001.zip › animals-1297365-supplementary.pdf]

Supplementary table 1: Summary of products.

Product	Country of Origin	Form	Labelled species	Labelled lifestage
1	Italy	Extruded	Dog	Adult maintenance
2	Italy	Extruded	Dog	Adult maintenance
3	Italy	Extruded	Cat	Adult maintenance
4	Italy	Canned	Dog	Adult maintenance
5	Italy	Canned	Dog	Adult maintenance
6	United Kingdom	Extruded	Dog	Adult maintenance
7	United Kingdom	Extruded	Dog	Adult maintenance
8	United Kingdom	Extruded	Dog	Growth and development
9	United Kingdom	Extruded	Cat	Adult maintenance
10	United Kingdom	Canned	Cat and Dog	Adult maintenance
11	United Kingdom	Tray	Dog	Adult maintenance
12	USA	Extruded	Dog	All life stages
13	USA	Extruded	Dog	All life stages
14	USA	Extruded	Dog	All life stages
15	USA	Extruded	Cat	All life stages
16	USA	Extruded	Cat	All life stages
17	USA	Canned	Cat and Dog	All life stages
18	USA	Canned	Cat and Dog	All life stages
19	Canada	Extruded	Dog	Adult maintenance
20	USA	Extruded	Dog	Adult maintenance
21	USA	Canned	Dog	Adult maintenance
22	USA	Extruded	Dog	Adult maintenance
23	USA	Canned	Dog	Adult maintenance
24	Canada	Extruded	Dog	All life stages
25	Canada	Extruded	Cat	All life stages
26	Canada	Extruded	Dog	Adult maintenance
